# Modified CAR T cells targeting membrane-proximal epitope of mesothelin enhances the antitumor function against large solid tumor

**DOI:** 10.1038/s41419-019-1711-1

**Published:** 2019-06-17

**Authors:** Zhiwei Zhang, Duqing Jiang, Huan Yang, Zhou He, Xiangzhen Liu, Wenxia Qin, Linfang Li, Chao Wang, Yang Li, He Li, Hai Xu, Huajun Jin, Qijun Qian

**Affiliations:** 1 Department of Biotherapy, The Eastern Hepatobiliary Surgery Hospital, Navy Medical University (Second Military Medical University), Shanghai, 201805 China; 2 Shanghai Engineering Research Center for Cell Therapy, Shanghai, 201805 China; 3 Departments of Respiratory and Critical Care Medicine, Changhai Hospital, Navy Medical University (Second Military Medical University), Shanghai, 200433 China

**Keywords:** Cancer immunotherapy, Drug delivery, Immunization

## Abstract

Mesothelin (MSLN) is an attractive antigen for chimeric antigen receptor (CAR) T therapy and the epitope selection within MSLN is essential. In this study, we constructed two types of CARs targeting either region I of MSLN (meso1 CAR, also known as a membrane-distal region) or region III of MSLN (meso3 CAR, also known as a membrane-proximal region) using a modified piggyBac transposon system. We reported that, compared with meso1 CAR T cells, meso3 CAR T cells express higher levels of CD107α upon activation and produce increased levels of interleukin-2, TNF-α, and IFN-γ against multiple MSLN-expressing cancer cells in vitro. In a real-time cell analyzer system and a three-dimensional spheroid cancer cell model, we also demonstrated that meso3 CAR T cells display an enhanced killing effect compared with that of meso1 CAR T cells. More importantly, in a gastric cancer NSG mice model, meso3 CAR T cells mediated stronger antitumor responses than meso1 CAR T cells did. We further identified that meso3 CAR T cells can effectively inhibit the growth of large ovarian tumors in vivo. Collectively, our study provides evidences that meso3 CAR T-cell therapy performs as a better immunotherapy than meso1 CAR T-cell therapy in treating MSLN-positive solid tumors.

## Introduction

Chimeric antigen receptor (CAR) T-cell therapy is an immunotherapeutic strategy which genetically modifies T cells expressing a cell-surface antigen to promote T-cell function and persistence. The remarkable efficacy of CD19-CAR T against hematologic malignancies encourages the exploration of CAR T therapies in solid tumors.^1–3^

A majority of CAR T studies utilize viral vectors to deliver CAR transgenes into T cells, owing to the high efficiency^4–7^. However, viral vector-based CAR T products require testing for the replication-competent virus to ensure safety, thereby leading to a complicated manufacturing process and high cost. Alternatively, non-viral vector piggyBac (PB) transposon can integrate CAR genes into T cells by electroporation, which is cost-effective, simple to use and no infectious risk^8,9^.

To generate high efficiency of CAR T cells, it is crucial to choose appropriate antigens to eliminate tumor cells with minimum toxicity. Mesothelin (MSLN) is becoming a promising antigen, because of its low expression on normal tissues and high expression on various solid tumors^10^. MSLN is a cell-surface glycoprotein with normal expression in peritoneum, pleura, and pericardium, but with overexpression in a variety of cancers, including mesothelioma, pancreatic, lung, gastric, and ovarian cancers^10,11^. Aberrant expression of MSLN plays a central role in cancer cell proliferation, invasion and metastasis through activating PI3K, ERK, and MAPK signaling pathways^12^. In contrast, the function of MSLN in healthy tissues is dispensable^13^, because of which a favorable safety profile had been observed in MSLN-targeted immunotherapies^14–16^. Thus, MSLN is an ideal cancer antigen for targeted immunotherapy^17,18^.

MSLN is a cell-surface glycoprotein, and its extracellular domain comprises of region I (residues 296–390), II (residues 391–486), and III (residues 487–598)^10,19^. The N-terminal region I and C-terminal region III correspond to the membrane-distal region (MDR) and membrane-proximal region (MPR) individually. Most of the current MSLN-based immunotherapies target the MDR of MSLN^17,20^, which also interacts with other functional proteins especially with CA125/MUC16^21^. Although the CAR T-cell therapies that target MDR of MSLN have been reported to inhibit cancer growth, the overall efficacy is still relatively low in vivo^18,22,23^. The low antitumor effect of CAR T cells may be caused by the MDR epitope that interacting with other functional proteins^24,25^. In addition, a recent study suggested that a novel monoclonal antibody targeting the MPR of MSLN was efficacious for solid tumors^19^. However, the direct functional comparison between MDR and MPR based immunotherapies has yet been identified. Therefore, we developed meso1 CAR T and meso3 CAR T targeting MDR and MPR of MSLN using the non-viral PB system, and the antitumor effects of the modified T cells were examined both in vitro and in vivo.

## Results

### Generation and characterization of meso1 CAR T and meso3 CAR T cells

**Fig. 1:**
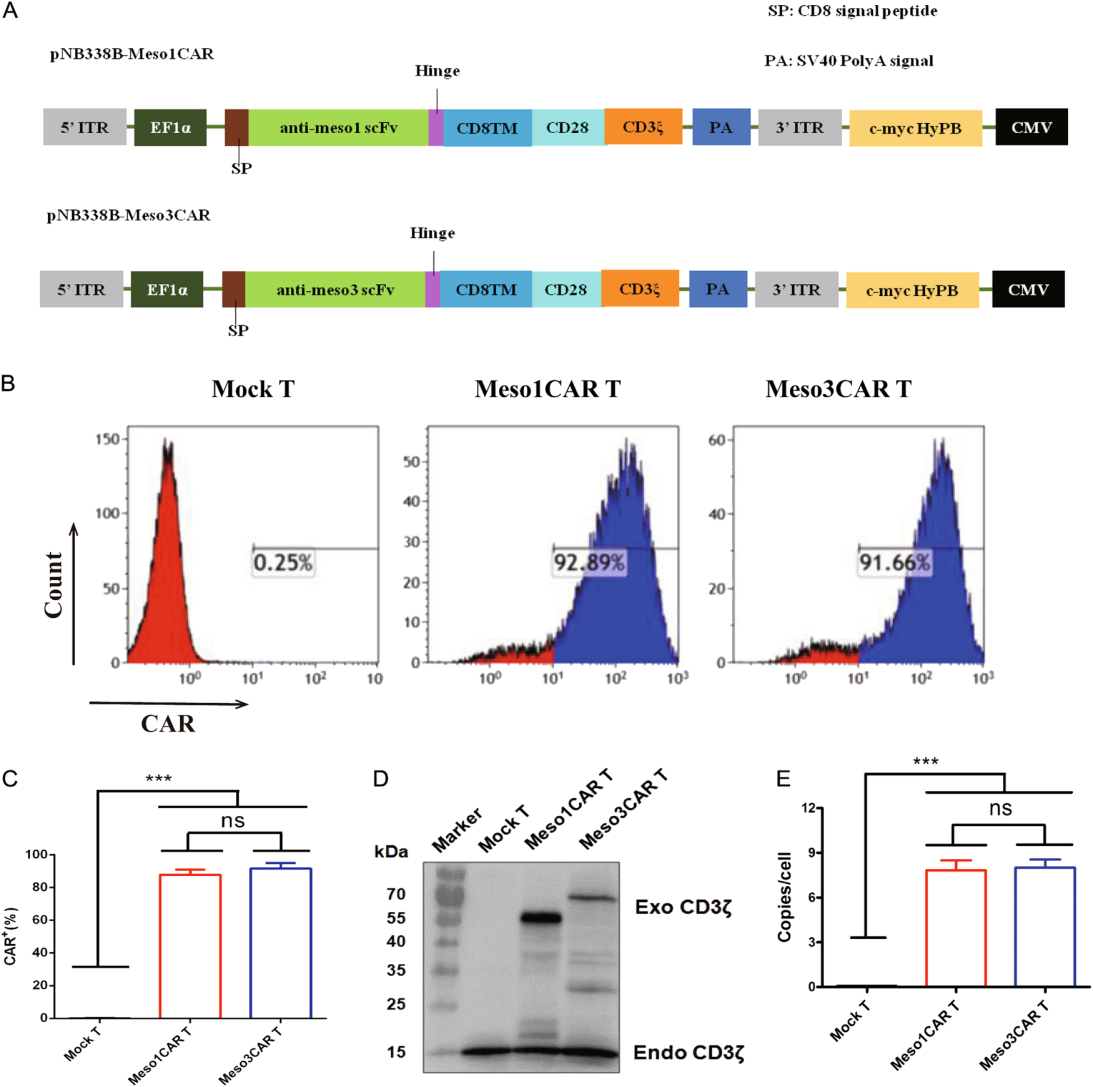
Generation and characterization of meso1 CAR T and meso3 CAR T cells. **a** Schematic illustration of the meso1 CAR and meso3 CAR constructs. **b** Positive ratio of meso1 CAR T and meso3 CAR T cells were detected by flow cytometry. **c** Statistic analysis of positive rate of CAR T cells. **d** The protein expression of exogenous CD3ζ was detected by western blotting. **e** The average copy-number of CAR in meso1 CAR T cells and meso3 CAR T cells was detected by RT-PCR. Data are expressed as the mean ± SD; *n* = 3; ns *p* > 0.05, ***p < 0.001

In order to generate the MSLN-CAR T cells, two CAR vectors targeting either region I of MSLN or region III of MSLN were constructed using a modified PB transposon system (Fig. 1a). Previous studies had demonstrated the PB system was an optimal method to deliver genes into T cells^26,27^. We modified it to improve the efficiency of positive cell enrichment by using MSLN peptide coated plates. Flow cytometry showed that the mean positive ratios of both CARs were above 80% (*n* = 3; 87.85% vs 91.75%, Fig. 1b, c). The CAR expression was further confirmed by Western blotting (Fig. 1d). In addition, the CAR genes were integrated into the genome of T cells at an average of 7–8 copies/cell (Fig. 1e). Collectively, these data display the successful construction of meso1 CAR T and meso3 CAR T cells.

### Contrastive analysis of meso1 CAR T and meso3 CAR T cells in vitro

**Fig. 2:**
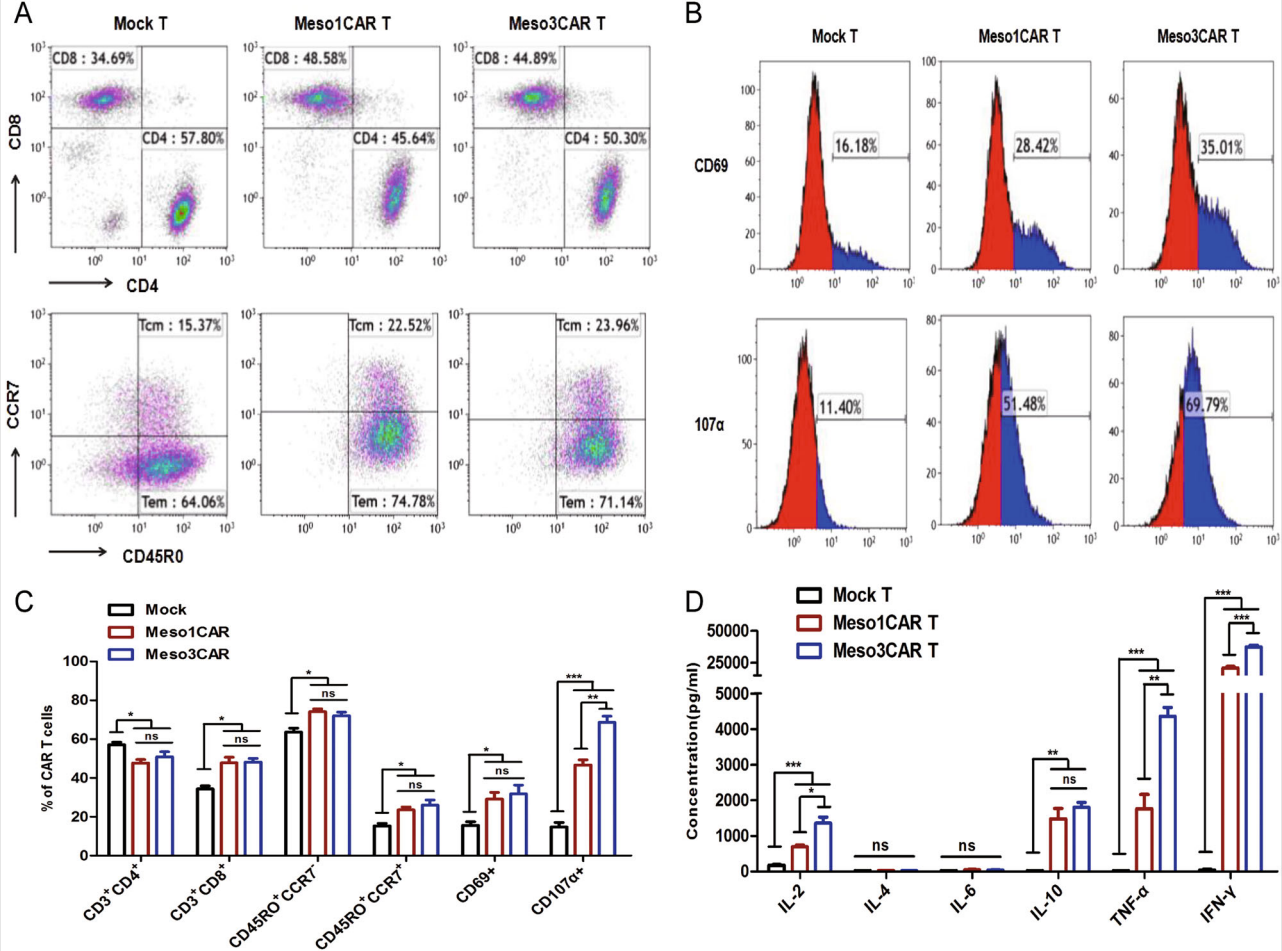
Contrastive analysis of the immunological activity of meso1 CAR T and meso3 CAR T cells. **a** CD3, CD4, CD8, and memory T cells of CAR T cells were assessed. Memory T cells contain central memory T cells (CD45RO^+^CCR7^+^) and effector memory T cells (CD45RO^+^CCR7^−^). **b** The activation markers CD69, and CD107α are detected. **c** Statistic analysis of T-cell markers. **d** Cytokines (IL-2, IL-4, IL-6, IL-10, TNF-α, and IFN-γ) were detected by flow cytometry using the CBA method. Data are expressed as the mean ± SD; *n* = 3; ns *p* > 0.05, *p < 0.05, **p < 0.01, ***p < 0.001

To test the immunocompetence of CAR T cells, T-cell markers, and cytokines were measured. Flow cytometry data indicated the mean percentage of CD3^+^CD8^+^ cells was significantly higher in both CAR T cells as compared with that in mock T cells (*n* = 3; 47.79% and 48.23% vs 34.33%, Fig. 2a, c). Also, the ratios of memory T cells in both CAR T cells were higher than that in control cells (Fig. 2a, c). In addition, the mean levels of activation marker CD69 were upregulated in both CAR T cells (*n* = 3; 29.11% and 31.80% vs 15.56%, Fig. 2b, c). Interestingly, although the levels of the lethality marker CD107α in the two CAR T groups were comparatively higher than that in mock T cells, the mean proportion of CD107α in meso3 CAR T cells showed much higher amount than that in meso1 CAR T cells (*n* = 3; 68.60% vs 46.69%, *p* < 0.01, Fig. 2b, c). The surface marker PD-1 was also tested and showed that the mean PD-1 expression was higher in meso1 CAR T cells and meso3 CAR T cells than that in mock T cells (27.59% and 31.44% vs 10.46%, *p* < 0.001, Fig. S1).

The proliferation of T cells was analyzed by Hoechst 33342/Ki-67 assay, showing that mean T cells at the S/G2/M phase increased in meso1 CAR T and meso3 CAR T groups as compared with that in the control group (*n* = 3; 39.09% and 40.70% vs 24.03%, *p* < 0.01, Fig. S2). These data indicate both CAR T cells exhibit stronger proliferation capacities. The immunologic activities of CAR T cells were also tested by cytokines. The levels of interleukin-2 (IL-2), IL-10, TNF-α, and IFN-γ secreted in both CAR T cells were significantly higher than that in the control T cells. Compared with meso1 CAR T cells, meso3 CAR T cells induced higher levels of IL-2, TNF-α, and IFN-γ (Fig. 2d). Taken together, compared with meso1 CAR T cells, meso3 CAR T cells express higher levels of CD107α upon activation and produce increased levels of IL-2, TNF-α, and IFN-γ cytokines against MSLN-positive tumor cells.

### Cytotoxicity of meso1 CAR T and meso3 CAR T cells against MSLN^+^ cancer cell lines in vitro

**Fig. 3:**
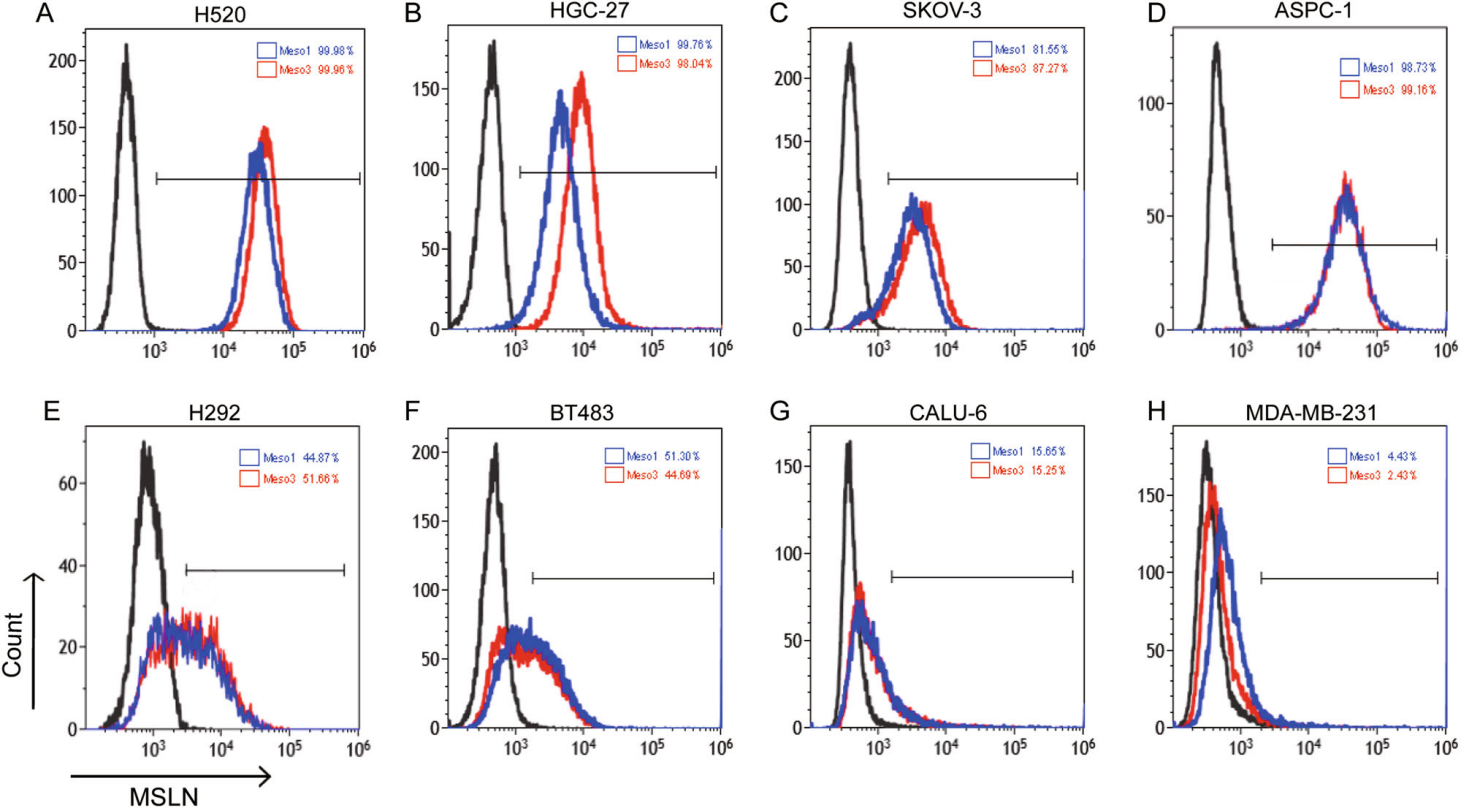
The expression of MSLN in cancer cell lines was detected by flow cytometry. Meso1 CAR-Fc and meso3 CAR-Fc were used as primary antibodies, whereas goat anti-human IgG Fc-PE was used as secondary antibody. **a** MSLN was expressed in H520. **b** MSLN was expressed in HGC-27. **c** MSLN was expressed in SKOV-3. **d** MSLN was expressed in ASPC-1. **e** MSLN was expressed in H292. **f** MSLN was expressed in BT483. **g** MSLN was expressed in CALU-6. **h** MSLN was expressed in MDA-MB-231

To select suitable cancer cell lines with high expression of MSLN, a panel of cell lines were analyzed by flow cytometry (Fig. 3). The results indicated that MSLN was highly expressed in H520, HGC-27, SKOV-3, ASPC-1, H292, and BT483 cells; but with low expression in CALU-6 and MDA-MB-231 cells. Therefore, HGC-27 and SKOV-3 cells were selected as targeted cell lines. To further confirmed MSLN expression in tissue levels, tissue chips from gastric cancer and ovarian cancer were measured by IHC (Fig. S3–5, Table S1). MSLN expressed mostly on cytomembrane, with limited expression in cytoplasm. In gastric cancer, MSLN expression in the meso3 group was higher than that in the meso1 group (46.6% vs 25.0%, *p* < 0.001). In ovarian cancer, a similar positive rate was detected in the two groups (55.8% vs 54.7%). In summary, these results confirm that MSLN is highly expressed in HGC-27 and SKOV-3 cell lines as well as in the corresponding cancer tissues.

**Fig. 4:**
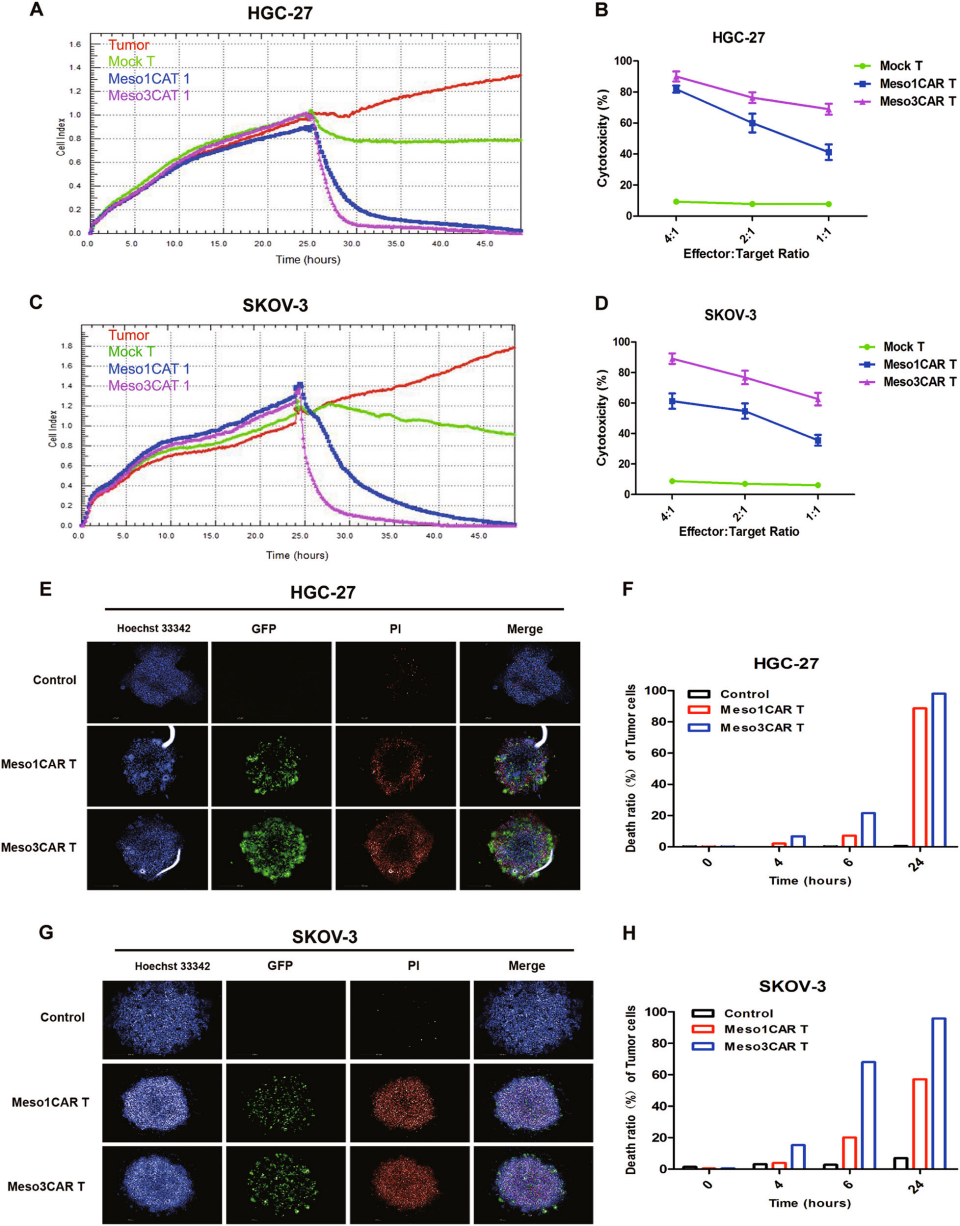
Meso3 CAR T cells are more cytotoxic than meso1 CAR T cells in vitro. **a** Cytotoxic activities of the meso1 CAR T cells and the meso3 CAR T cells against HGC-27 cells were measured using RTCA system at the E:T ratio of 2:1. **b** Quantified data on the specific lytic levels of CAR T cells against HGC-27 cells were analyzed at different E:T ratios (mean ± SD; *n* = 3; one way ANOVA test). **c** Cytotoxic activities of the meso1 CAR T cells and the meso3 CAR T cells against SKOV-3 cells were measured using RTCA system at the E:T ratio of 2:1. **d** Quantified data on the specific lytic levels of CAR T cells against SKOV-3 cells were analyzed at different E:T ratios (mean ± SD; *n* = 3; one way ANOVA test). **e** The killing activity of meso1 CAR and meso3 CAR T cells was detected using the 3D cancer spheroid model in gastric cancer. **f** The time effect of death rate of tumor cells was shown by histogram in gastric cancer. **g** The killing activity of meso1 CAR and meso3 CAR T cells was detected using the 3D cancer spheroid model in ovarian cancer. **h** The time effect of death rate of tumor cells was shown by histogram in ovarian cancer

In cytotoxicity assays, the results revealed that meso3 CAR T cells exhibited stronger cytotoxicity than meso1 CAR T cells in a dose-dependent manner (Fig. 4a–d). At the low ratio of 1:1, the cytotoxicity of meso3 CAR T cells is stronger than that of meso1 CAR T cells (*n* = 3; 68.9% vs 41.2% against gastric cancer, *p* < 0.001; 62.6% vs 35.6% against ovarian cancer, *p* < 0.001). Then, we generated MSLN knockdown SKOV-3 cells using shMSLN and control cells with shCtrl. Results showed that meso1 CAR T cells and meso3 CAR T cells had stronger cytotoxicity against SKOV-3 cells transduced with shCtrl, while got weak cytotoxicity for SKOV-3 cells with MSLN knockdown (Fig. S6). Furthermore, in the three-dimensional (3D) cancer spheroid model, although both CAR T cells were found to infiltrate into the tumor sphere, meso3 CAR T cells were more lethal to cancer cells than meso1 CAR T cells (Fig. 4e–h). Collectively, these results above indicate that meso3 CAR T cells exhibit greater cytotoxicity against MSLN-positive cancer cells than meso1 CAR T cells in vitro.

### Antitumor activities of meso1 CAR T and meso3 CAR T cells against MSLN^+^ gastric cancer in vivo

**Fig. 5:**
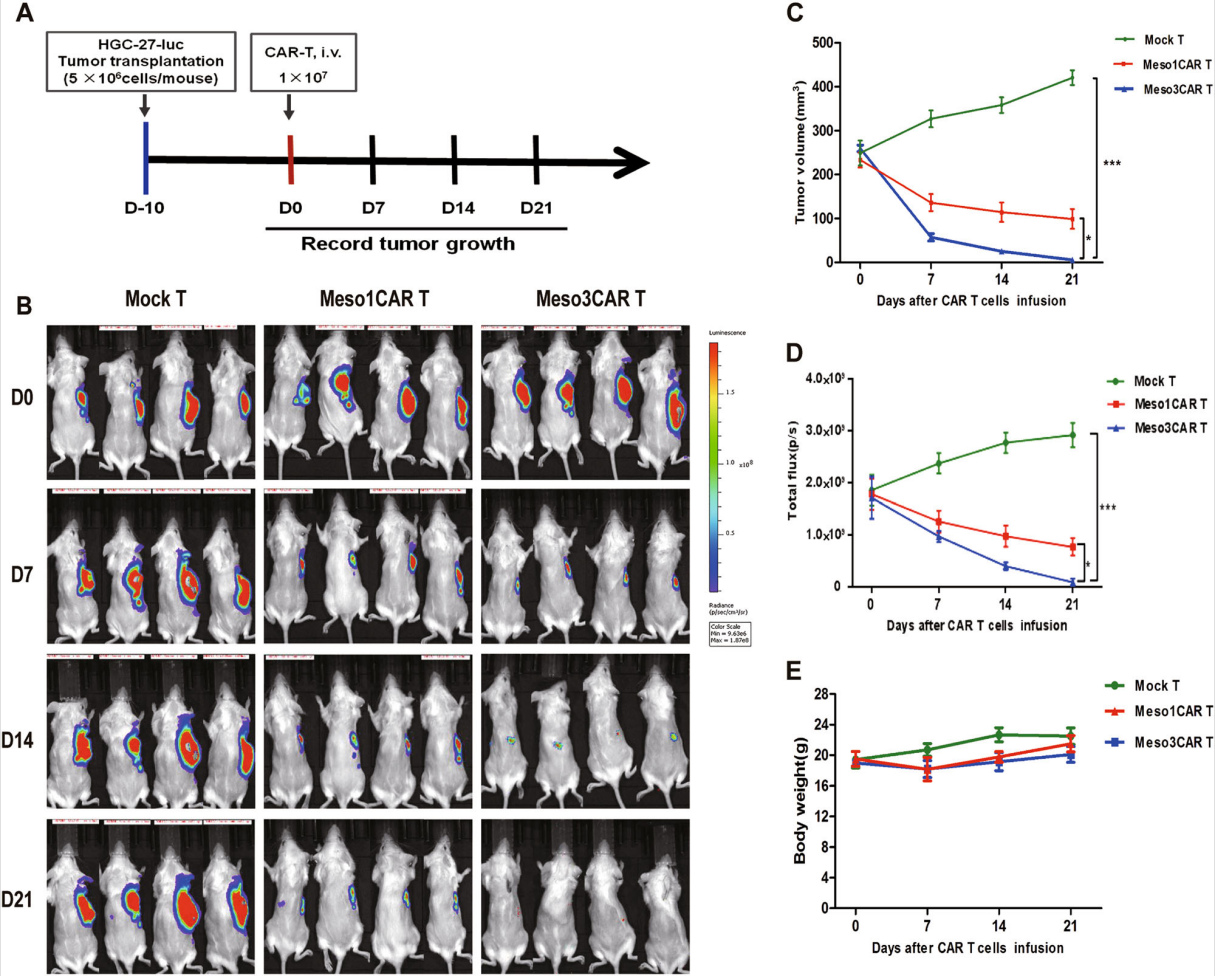
Therapeutic efficacy of meso1 CAR T cells and meso3 CAR T cells against gastric cancer in vivo. **a** Schematic representation of the procedure in gastric cancer. **b** Imaging scans of mice during the treatment in HGC-27 cancer model. **c** Tumor volume was measured and analyzed. **d** Fluorescence intensity of cancer cells was detected using an in vivo imaging system. **e** Body weight was recorded and analyzed. Data are expressed as the mean ± SD; *n* = 4 per group; *p < 0.05,***p < 0.001

To determine the efficacy in vivo, the antitumor responses of CAR T cells in HGC-27 xenograft mouse model were examined. It was shown that the average tumor volume in the mock T group was increased to 420.4 mm^3^, whereas that in meso1 CAR T group was 98.8 mm^3^. Interestingly, tumors were almost eliminated in meso3 CAR T group (Fig. 5a–c), which is consist with the total fluorescence intensity data showing that the fluorescence intensity in meso3 CAR T group was significantly reduced compared with meso1 CAR T group (Fig. 5d). No differences in body weight among these three groups were observed (Fig. 5e). The potential organ toxicity was examined using hematoxylin and eosin (HE) staining, which revealed that no organ toxicities were found in normal tissues (Fig. S7), indicating that meso3 CAR T cells are of safety with no serious off-target toxicity. Thus, the results indicate that meso3 CAR T cells display a stronger antitumor response than that of meso1 CAR T cells in vivo, and no harm to normal tissue.

### Antitumor activities of Meso3 CAR T cells against large established ovarian cancer in vivo

**Fig. 6:**
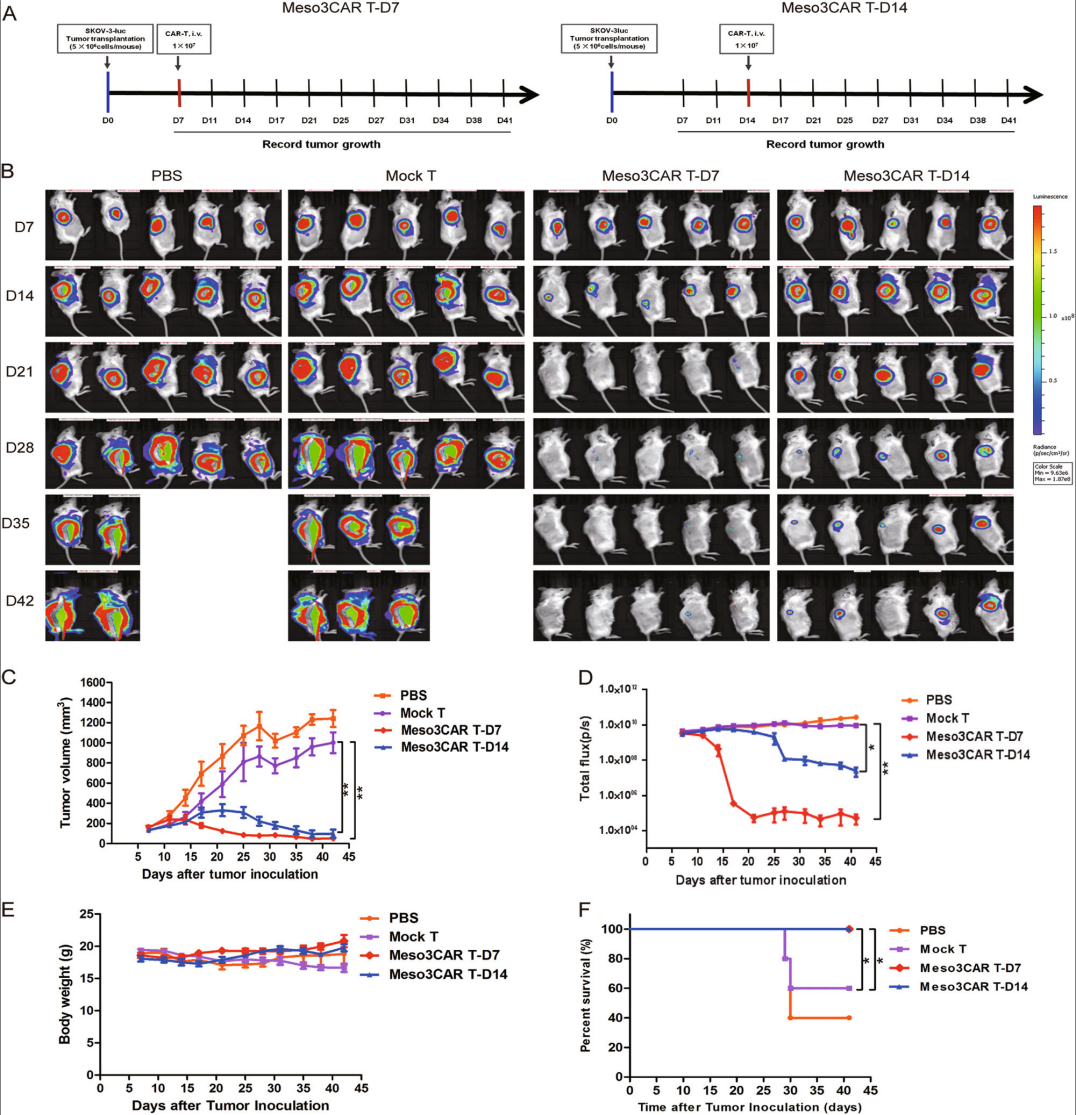
Efficacy of meso3 CAR T-cell therapy against large established ovarian cancer in vivo. **a** Schematic representation of the procedure in ovarian cancer. **b** Imaging scans of mice during treatment in the SKOV-3 cancer model. **c** Tumor volume was measured and analyzed. **d** Tumor fluorescence intensity was detected using an in vivo imaging system. **e** Body weight was recorded. **f** The survival time was analyzed. Data are expressed as the mean ± SD; *n* = 5 per group; ns *p* > 0.05, *p < 0.05, **p < 0.01, ***p < 0.001

As meso3 CAR T cells had shown better antitumor activities in the gastric cancer model, it was sought to further explore their efficacy in an ovarian cancer mouse model with large established tumors (Fig. 6a). It was shown that meso3 CAR T cells eliminated most of the ovarian tumors in the early treatment group. Importantly, in the late treatment group, meso3 CAR T cells showed an efficient inhibition of the growth of large established tumors, and got no effect on body weight (Fig. 6b–e). Furthermore, the survival time of mice in meso3 CAR T early and late treatment groups was significantly longer as compared to that in the mock T group (Fig. 6f). Taken together, results above show that meso3 CAR T cells exhibit excellent antitumor response against the early stage of ovarian cancer, and a promising antitumor activity against the large ovarian cancer.

## Discussion

In this study, we generated Meso1 CAR and Meso3 CAR targeting to MDR and MPR epitopes of MSLN, respectively, using the non-viral PB transposon system. Comparing with Meso1 CAR, Meso3 CAR produces higher levels of cytokines as well as eliciting stronger cytotoxicities against MSLN-expressing cancer cells in vitro and in a gastric cancer xenograft mouse model. Therefore, we suggest that MSLN MPR is a better epitope to target than MDR for CAR T design. We further provide evidence that Meso3 CAR T cells efficiently suppresses the large ovarian tumors in an NGS mouse model. Considering MSLN is an attractive solid tumor antigen, our work that identifies the better epitope for MSLN CARs contributes to the future clinical application for MSLN-positive patients.

Although MSLN has been targeted in a few CAR T therapies, the antitumor activities showed in these studies are modest. In a PDX model of pancreatic cancer, tumor only had slight regression when treated with MSLN-CAR T cells^18^. Another study had two patients with solid malignancies infused with MSLN-CAR T cells multiple times, both of them displayed stable disease^28^. They also prove the safety and antitumor activity of MSLN-CAR T cells in pancreatic ductal adenocarcinoma, in which two of six patients had stable disease^29^. These modest effects toward solid tumors clearly unmet the clinical request for improving responses. To be noted, all CARs used in the above studies targeted SS1 domain in MSLN region I (296–390), which belongs to the MDR. Here, our study provides an alternative MSLN epitope for CARs, which was proven to have a better antitumor performance in vivo.

The MDR of MSLN is the binding domain for some functional proteins such as CA125/MUC16. In this case, antibody-based products targeting the region I have to compete with CA125/MUC16 for the MSLN antigen interaction, which may weaken the binding and function of these therapeutic reagents. For the same consideration, a previous study developed an MSLN region III (MPR)-targeted immunotoxin and showed its potent antitumor activities in vivo^19^.

Another explanation for the better antitumor effect observed in meso3 CAR could be due to the specific characteristics of the MPR epitope. As the MPR bridges the extracellular domain and transmembrane region of MSLN, it is the region where might have a rigid structure or responsible for a specific function, which is to provoke stronger antitumor response. A similar mechanism has been applied to HIV vaccine design, in which the MPR of gp41 was considered as the key factor to cause stronger immune responses^30,31^.

Reportedly, increased levels of the degranulation marker CD107α positively correlated with the function of T cells^32^. The present study also shows that CD107α in meso3 CAR T cells was highly expressed as compared with that in mock T and meso1 CAR T cells. Moreover, IFN-γ production was significantly increased in the meso3 CAR T cells accompanied with high level of IL-2 and TNF-α, which exerted a synergistic enhancing effect for CAR T-cell function. This finding is agreed with a previous study, which found that IFN-γ production results in strong cytotoxicity of T cells^33^.

The primary advantages of the PB transposon system are high safety and easy to manufacture, making it suitable for clinical translation of CAR T-cell therapy^8,9^. Moreover, the PB transposon system is adaptable for large and multiple gene fragments. However, its transfection efficiency is relatively low in T cells, averaging from 20–70%^34,35^. In this study, we optimized the PB transfection protocol and developed an MSLN-coated plate cell enrichment method, by which positive CAR T cells can be above 80%, making it adequate for the clinical application.

The treatment of large tumors using CAR T cells is usually challenging. Carpenito et al.^36^ successfully treated large tumors (≈ 500 mm^3^) using lentiviral vector engineered MSLN-CAR T cells which contain costimulatory domains of CD28 and CD137 (4–1BB). Another study documented that the growth of large solid tumors can be repressed using PD-1-CAR T cells, owing to increased CAR T-cell infiltration and reduced inhibitory PD-1 signaling^37^. The present study shows that meso3 CAR T cells can significantly inhibit large tumor growth (≈ 350 mm^3^), suggesting that targeting MPR epitope of MSLN is also a promising treatment for MSLN-expressing cancers.

For CAR design in solid tumors, two of co-stimulating domains, 4–1BB and CD28, are most widely used. Some researchers have adopted 4–1BB as costimulatory domain, which can get persistent therapeutic effect with minor side effects^4,38^, other researchers have utilized CD28, which can get stronger antitumor effect^39,40^. A study has indicated that CD28 or 4–1BB costimulation can lead into distinct signaling in glycometabolism, 4–1BB costimulation enhances mitochondrial oxidative phosphorylation of CAR T cells, whereas CD28 costimulation improves glycolysis to meet their metabolic demands^41^. Given the hypoxic microenvironment of solid tumors, CD28 costimulatory domain may benefit better than 4–1BB for CAR T cells to survive within solid tumors. Moreover, a common advantage of non-viral systems relative to viral systems is that the obtained CAR T cells can persist a higher level of memory T cells^42,43^. Taken together, use of the non-viral system mediated by piggyBac transposon and adoption of CD28 costimulatory domain might be an ideal option to improve persistence of CAR T cells both in vitro and in vivo.

Although meso3 CAR T cells showed safety and therapeutic advantage in gastric cancer and ovarian cancer, there are still several limitations. First, a subset of MSLN-negative cancer cells limits the cytotoxic effect of meso3 CAR T cells. Second, the tumor microenvironment might reduce the efficacy of CAR T cells. For improving the CAR T cells effect, combined immunotherapies are becoming a new research field. PD-1 blocking antibodies were used in combination with CAR T-cell therapy to improve the antitumor effect^44,45^. Another strategy is that the CAR T cells are engineered to express the checkpoint inhibitors, which also gets promising efficacy for advanced solid tumors^37,46^.

In summary, the present study demonstrates meso3 CAR T cells targeting MPR of MSLN exhibit a strong efficacy against MSLN-expressing tumors as compared to that of meso1 CAR T cells targeting MDR. Moreover, meso3 CAR T cells could effectively inhibit the growth of large tumors in vivo, suggesting that MPR of MSLN is a promising epitope to target for solid tumor. Although CAR T-cell therapy has shown its potential in solid cancers, the efficacy will be weakened facing to large tumor burdens, in which cases, combined immunotherapies are expected to have a better performance.

## Materials and methods

### Cell lines

Cancer cell lines were purchased from the Cell Bank of the Chinese Academy of Sciences (Shanghai, China), including H292, H520, and CALU-6 (lung cancer), BT483 and MDA-MB-231 (breast cancer), HGC-27 (gastric cancer), SKOV-3 (ovarian cancer), and ASPC-1 (pancreatic cancer). These cells were cultured in RPMI-1640 or DMEM medium containing 10% fetal calf serum at 37 °C with 5% CO2. The *ffluc/GFP* fusion gene was transduced into HGC-27 and SKOV-3 cells to establish the HGC-27-luc and SKOV-3-luc cells.

### Generation of modified CAR T cells

The *meso3 CAR* gene was cloned into the PB transposon vector pNB328-EF1α to construct pNB328-meso3 CAR. pNB328-meso1 CAR and empty pNB328 vectors were used as controls. The antibody sequence for Meso3 CAR T was derived from the YP218 antibody, which was originally discovered by the NIH (https://www.nature.com/articles/srep09928; US Patent: US9803022: https://patents.google.com/patent/US9803022). In addition, the antibody sequence for Meso1 CAR T was derived from the SS1 antibody, which was also originally discovered by the NIH (US Patent: US7081518: https://patents.google.com/patent/US7081518?oq=patent:7081518).

Fresh blood was collected from healthy volunteers after informed consent under a protocol approved by the Ethics Committee of the Second Military Medical University, China. For the generation of meso3 CAR T cells, peripheral blood mononuclear cells (PBMCs) were isolated, suspension cells were collected after adherent culture, then resuspended in electroporation buffer, and recombinant pNB328-meso3 CAR plasmids were electroporated into T cells according to the manufacturer’s instructions (Lonza, Switzerland). Then, the T cells transfected with MSLN-CAR or Mock plasmid were seeded in six-well plates, which had been coated with MSLN antigen (5 μg mL^−1^)/anti-CD28 antibody (5 μg mL^−1^) or anti-CD3 antibody (5 μg mL^−1^)/anti-CD28 (5 μg mL^−1^) antibody, respectively. The T cells were specifically stimulated with the antigens/antibodies for 3 days in medium containing 200 U/mL recombinant human IL-2. Thereafter, the activated cells were cultured in medium containing 100 U/mL IL-2. All modified T cells were maintained in the medium for 10–14 days to proliferate enough quantity of CAR T cells.

### Flow cytometry

The expression of MSLN on cancer cells was detected by flow cytometry, using meso1 CAR-Fc and meso3 CAR-Fc as primary antibodies followed by goat anti-human-PE secondary antibody (eBioscience, USA). The expression of CAR on CAR T cells was detected using MSLN-Fc-biotin, followed by staining with PE-streptavidin.

The immunophenotypes of T cells were tested using flow cytometry. Antibodies used for analysis include: CD3-PE-CY5, CD4-PE, CD8-FITC, and CD45RO-PE-CY5, CCR7-FITC, CD69-PC5, CD107α-PE-CY5, and PD-1-PE (BD Biosciences, USA). The proliferation of T cells was also assessed by flow cytometry. T cells were fixed using fixation/permeabilization solution kit, then incubated with Ki-67-APC and Hoechst 33342. All the data above were analyzed using the Kaluza analysis software (Beckman Coulter, USA).

### Immunohistochemistry (IHC)

The paraffin-embedded samples were sliced into 4-µm sections and baked at 70 °C for 2 h, followed by being deparaffinized in xylene and rehydrated in graded ethanol. The endogenous peroxidase was blocked, the antigen was retrieved, and blocked using goat serum. The sections were then probed with primary antibodies (biotinylated meso1 and meso3 antibodies), followed by horseradishperoxidase (HRP)-conjugated anti-biotin antibody. Subsequently, the slides were developed with DAB and counterstained with hematoxylin. Pancreatic cancer tissues served as the positive control for MSLN staining, whereas the pre-immune mouse IgG was used as the negative control.

### Generation of MSLN knockdown SKOV-3 cells

Knockdown of MSLN in the SKOV-3 cells and the mock vector control cells were generated through shRNA lentiviral vectors with two shMSLN and scrambled shRNA (Genechem, China), respectively, according to the manufacturer’s instructions. The lentiviral vectors and polybrene were added into the medium when the cells grew up to 30–40% confluence. 12 h later, fresh medium was replaced; then, transfected cells were selected with puromycin. The knockdown effect was verified by Western blotting and the cells were used for further experiments.

### Western blot analysis

Western blotting was performed as described previously^47^. T cells were harvested, lysed, and boiled to prepare the samples, subsequently separated by 10% sodium dodecyl sulfate polyacrylamide gel electrophoresis, and transferred to polyvinylidene difluoride membranes. After blocking, the membranes were probed with antibodies specific for CD3ζ or MSLN (Abcam, UK), followed by incubation with HRP-conjugated secondary antibodies. Protein bands was exposed to ECL (GE Healthcare, USA) followed by autoradiography. The endogenous CD3ζ or GAPDH was served as an internal control.

### Real-time PCR (RT-PCR)

T cells were collected, and total DNA was extracted using a gDNA extraction kit (Takara, Japan). The copies number of CAR genes was analyzed by RT-PCR. The reaction was carried out using a SYBR Green PCR Master Mix Kit (Toyobo, Japan) according to the manufacturer’s instructions. The relative expression level was normalized to that of β-actin and calculated using the 2^−ΔΔCt^ method.

### Cytokines assays

A total of 1 × 10^6^ CAR T cells were cultured in MSLN-coated plates for 24h. Then, cell supernatants were collected and treated using the Cytometric Bead Array (CBA) Human Th1/Th2 Cytokine Kit II according to the manufacturer’s instructions (BD, USA). The levels of IL-2, IL-4, IL-6, IL-10, TNF-α, and IFN-γ were measured by flow cytometry (Beckman Coulter, USA).

### Cytotoxicity assays

A real-time cell analyzer system (RTCA) and 3D spheroid cancer cell fluorescence measurements were used to assess the cytotoxicity. In the RTCA system, 1 × 10^4^ tumor cells/well were seeded and cultured for 24 h. Then, T cells were added into the unit at various effector/target cell (E:T) ratios (4:1, 2:1, and 1:1). The impedance signals were recorded at 5 min intervals. The signal-time curves were drawn to display the cytotoxicity.

In the 3D spheroid model, 5 × 10^3^ cancer cells were stained using Hoechst stain (Beyotime, China) and plated into each well for 48 h to generate 3D spheroid cancer cells. Then, the T cells were stained with Calcein-AM (Dojindo, Japan) and added to the wells at the E:T ratio of 2:1, together with propidium iodide. The fluorescence values were analyzed using high-intension confocal microscopy (Opera Phenix, PerkinElmer) at 0, 4, 6, and 24 h after co-culturation.

### In vivo experiments

Animal experiments were approved by the Institutional Animal Care and Use Committee of the Second Military Medical University, China. Female NSG mice, aged 4–6 weeks, were obtained from Beijing Vitalstar Biotechnology Co. Ltd (Beijing, China).

In the HGC-27-luc xenograft experiments, 5 × 10^6^ HGC-27-luc cells were subcutaneously injected into NSG mice. At 10 days post injection, 12 mice were randomly divided into three groups: mock T, meso1 CAR T, and meso3 CAR T. Then, mice were injected with 1 × 10^7^ T cells intravenously. Every 7 days, the fluorescent images were acquired using Xenogen IVIS imaging system (PerkinElmer, USA), and the tumor volume was calculated using the formula: *V* = ½ (length × width^2^); also, the body weight was analyzed.

In the SKOV-3-luc xenograft experiments, the same method was used to assess the antitumor effect. In all, 5 × 10^6^ SKOV-3-luc cells were subcutaneously injected into each mouse. Twenty mice were randomly divided into four groups: PBS, mock T, meso3 CAR T early treatment (a small tumor was established), and meso3 CAR T advanced treatment (a large tumor was established). Mice were intravenously injected with 100 μL PBS or 1 × 10^7^ meso3 CAR T cells/mouse on day 7 or 14. The tumor volume, fluorescent images, body weight, and survival were recorded and analyzed every 3–4 days.

### HE staining

The sections obtained from organs (including heart, liver, spleen, lung, kidney, and brain) from each group were paraffin-embedded and sliced into 4-µm sections. Then, the slides were baked at 65 °C for 1 h, deparaffinized in xylene, rehydrated by graded ethanol, and stained with HE successively.

### Statistical analysis

All data are presented as mean ± SD. Statistical significance was analyzed by Student’s *t* test, variance, or chi-square test. The survival data were analyzed by Kaplan–Meier curves and log-rank test. *p* < 0.05 were considered statistically significant. All analyses were performed using GraphPad Prism v7.0 (La Jolla, CA, USA).

## Supplementary information

Figure S1

Figure S2

Figure S3

Figure S4

Figure S5

Figure S6

Figure S7

Table S1

Supplementary figure legends
