# Correction: Modified CAR T cells targeting membrane-proximal epitope of mesothelin enhances the antitumor function against large solid tumor

**DOI:** 10.1038/s41419-020-2450-z

**Published:** 2020-04-16

**Authors:** Zhiwei Zhang, Duqing Jiang, Huan Yang, Zhou He, Xiangzhen Liu, Wenxia Qin, Linfang Li, Chao Wang, Yang Li, He Li, Hai Xu, Huajun Jin, Qijun Qian

**Affiliations:** 1 Department of Biotherapy, The Eastern Hepatobiliary Surgery Hospital, Navy Medical University (Second Military Medical University), Shanghai, 201805 China; 2 Shanghai Engineering Research Center for Cell Therapy, Shanghai, 201805 China; 3 Departments of Respiratory and Critical Care Medicine, Changhai Hospital, Navy Medical University (Second Military Medical University), Shanghai, 200433 China

**Keywords:** Cancer immunotherapy, Drug delivery, Immunization

Correction to: *Cell Death and Disease*

10.1038/s41419-019-1711-1

published online 17 June 2019

In the original published version of the article, the following information was omitted from the Methods section. The second paragraph should have read as follows:

**Generation of modified CAR T cells**

The *meso3 CAR* gene was cloned into the PB transposon vector pNB328-EF1α to construct pNB328-meso3 CAR. pNB328-meso1 CAR and empty pNB328 vectors were used as controls. The antibody sequence for Meso3 CAR T was derived from the YP218 antibody, which was originally discovered by the NIH (https://www.nature.com/articles/srep09928; US Patent: US9803022: https://patents.google.com/patent/US9803022). In addition, the antibody sequence for Meso1 CAR T was derived from the SS1 antibody, which was also originally discovered by the NIH (US Patent: US7081518: https://patents.google.com/patent/US7081518?oq=patent:7081518).

This has been corrected in both the PDF and HTML versions of the Article.

