# Supplementary figures and images for: Modified CAR T cells targeting membrane-proximal epitope of mesothelin enhances the antitumor function against large solid tumor
Source: Cell Death Dis. 2019 Jun 17;10(7):476. doi: 10.1038/s41419-019-1711-1 (PMC6572851; doi:10.1038/s41419-019-1711-1)

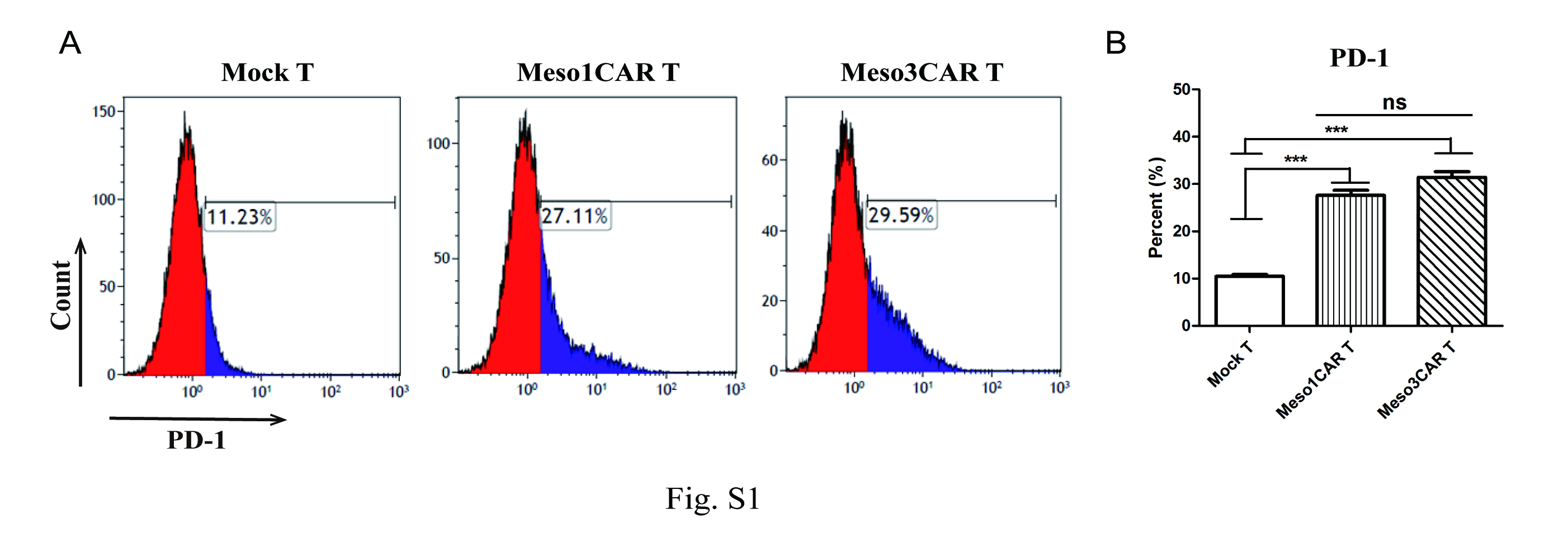

Supplement: Supplementary file 1 — Figure S1 [file 41419_2019_1711_MOESM1_ESM.tif]

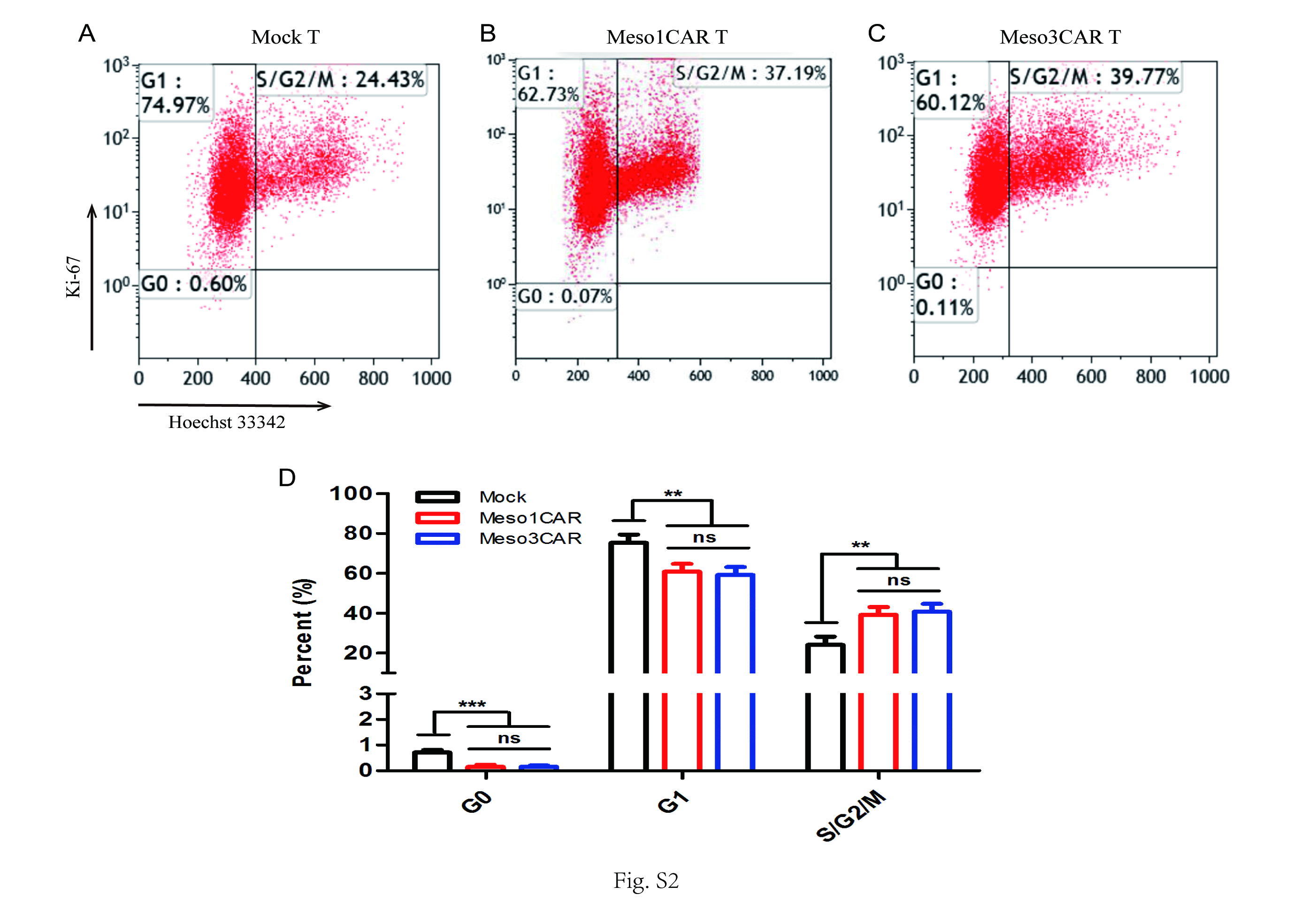

Supplement: Supplementary file 2 — Figure S2 [file 41419_2019_1711_MOESM2_ESM.tif]

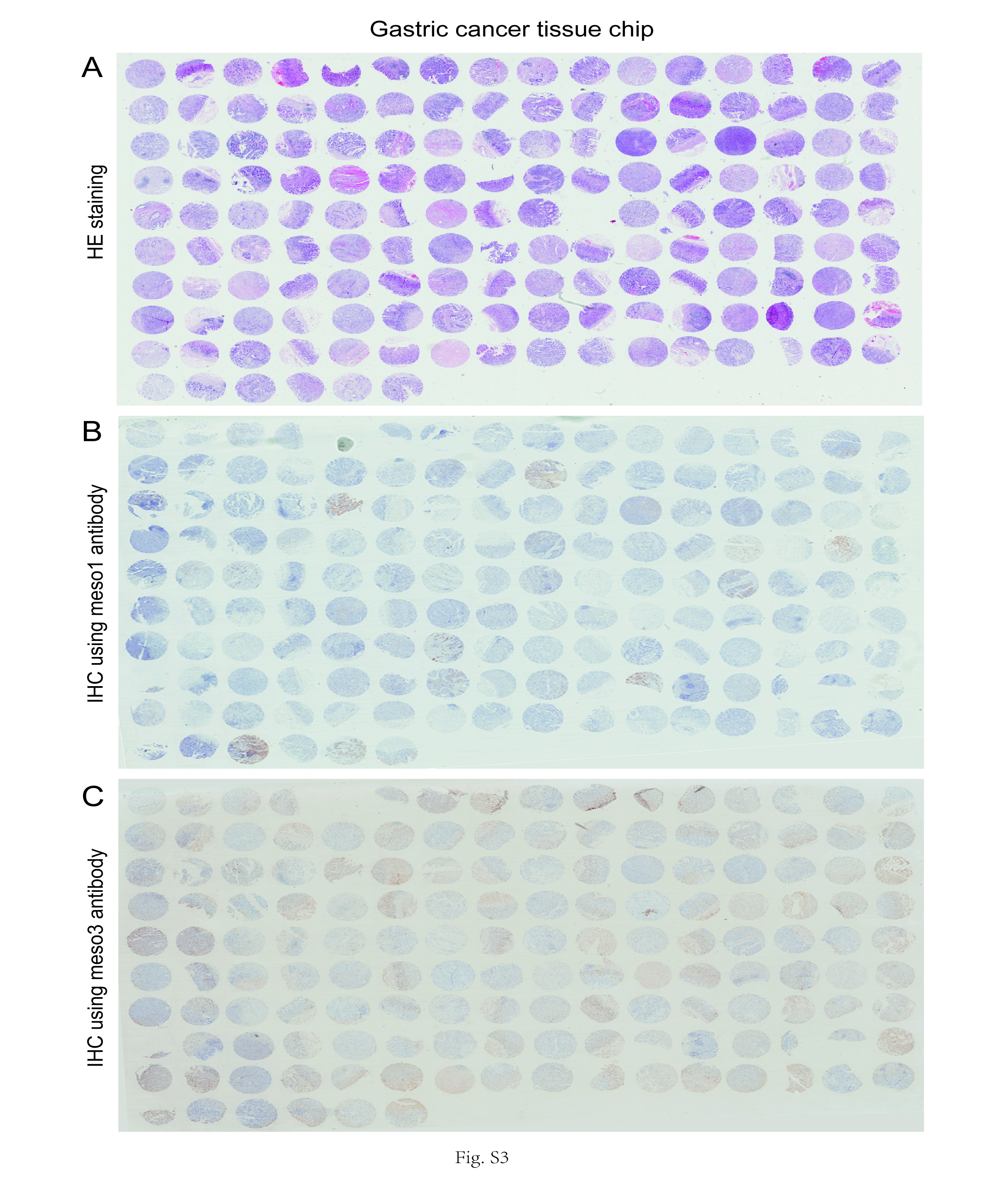

Supplement: Supplementary file 3 — Figure S3 [file 41419_2019_1711_MOESM3_ESM.tif]

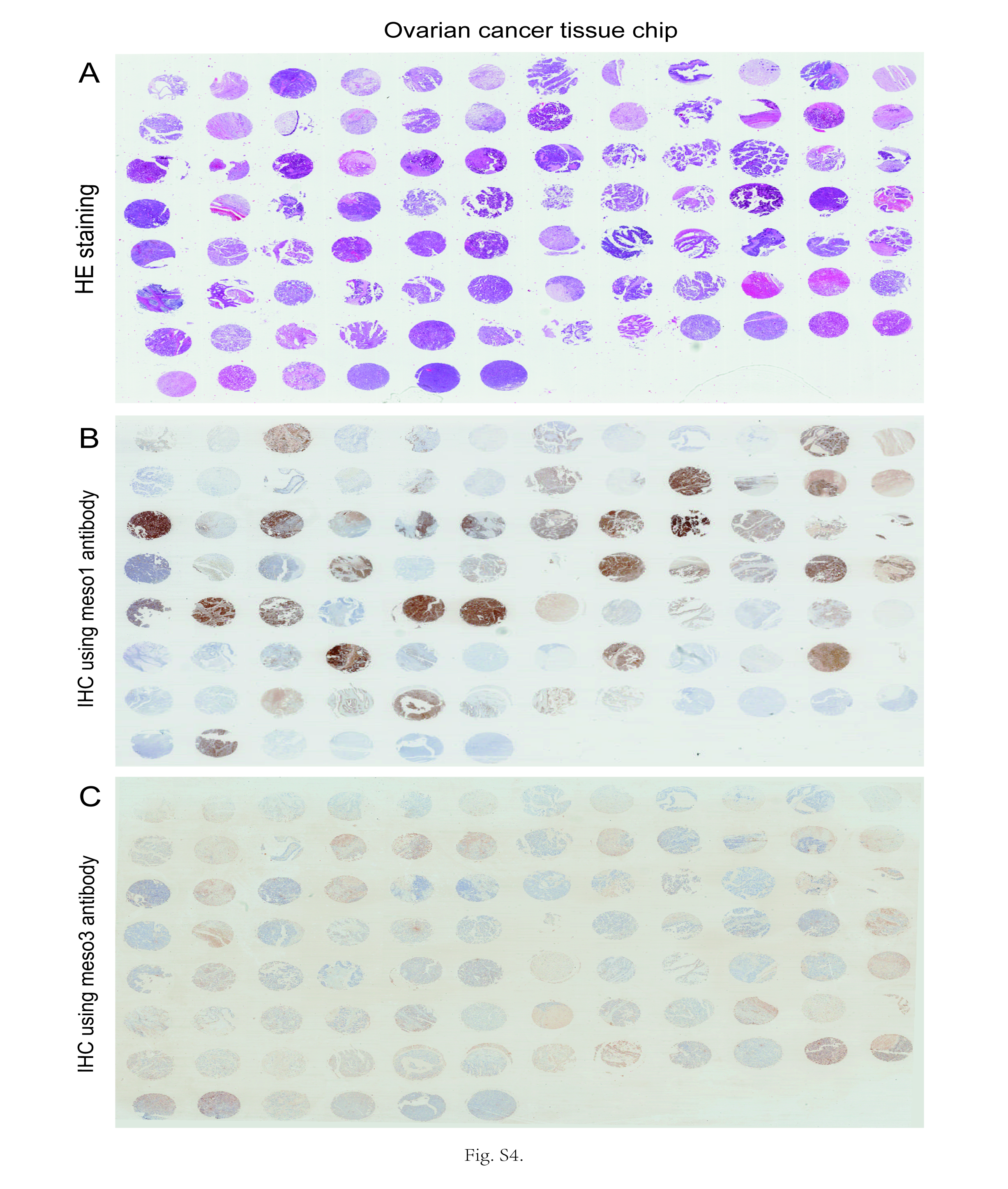

Supplement: Supplementary file 4 — Figure S4 [file 41419_2019_1711_MOESM4_ESM.tif]

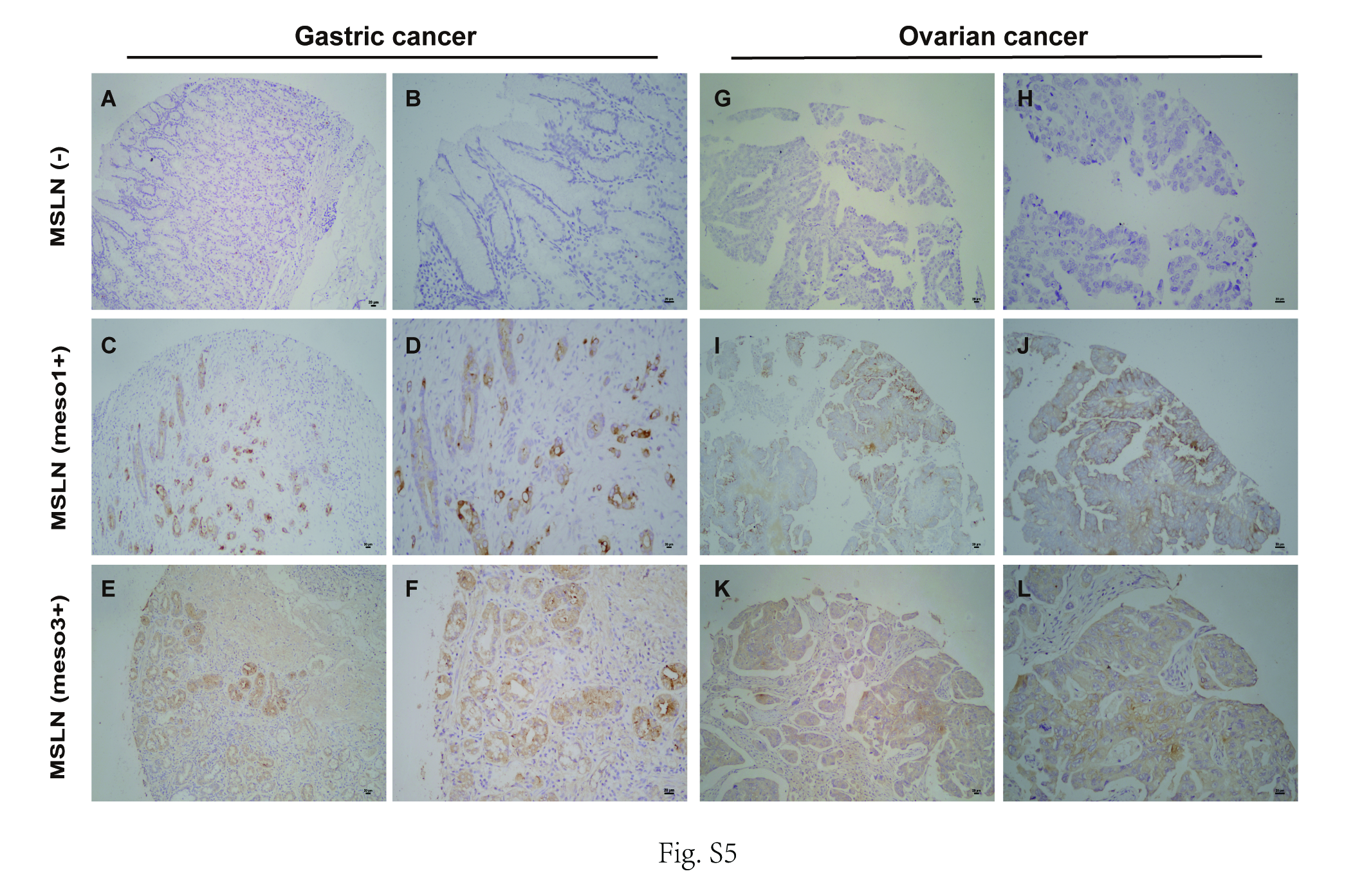

Supplement: Supplementary file 5 — Figure S5 [file 41419_2019_1711_MOESM5_ESM.tif]

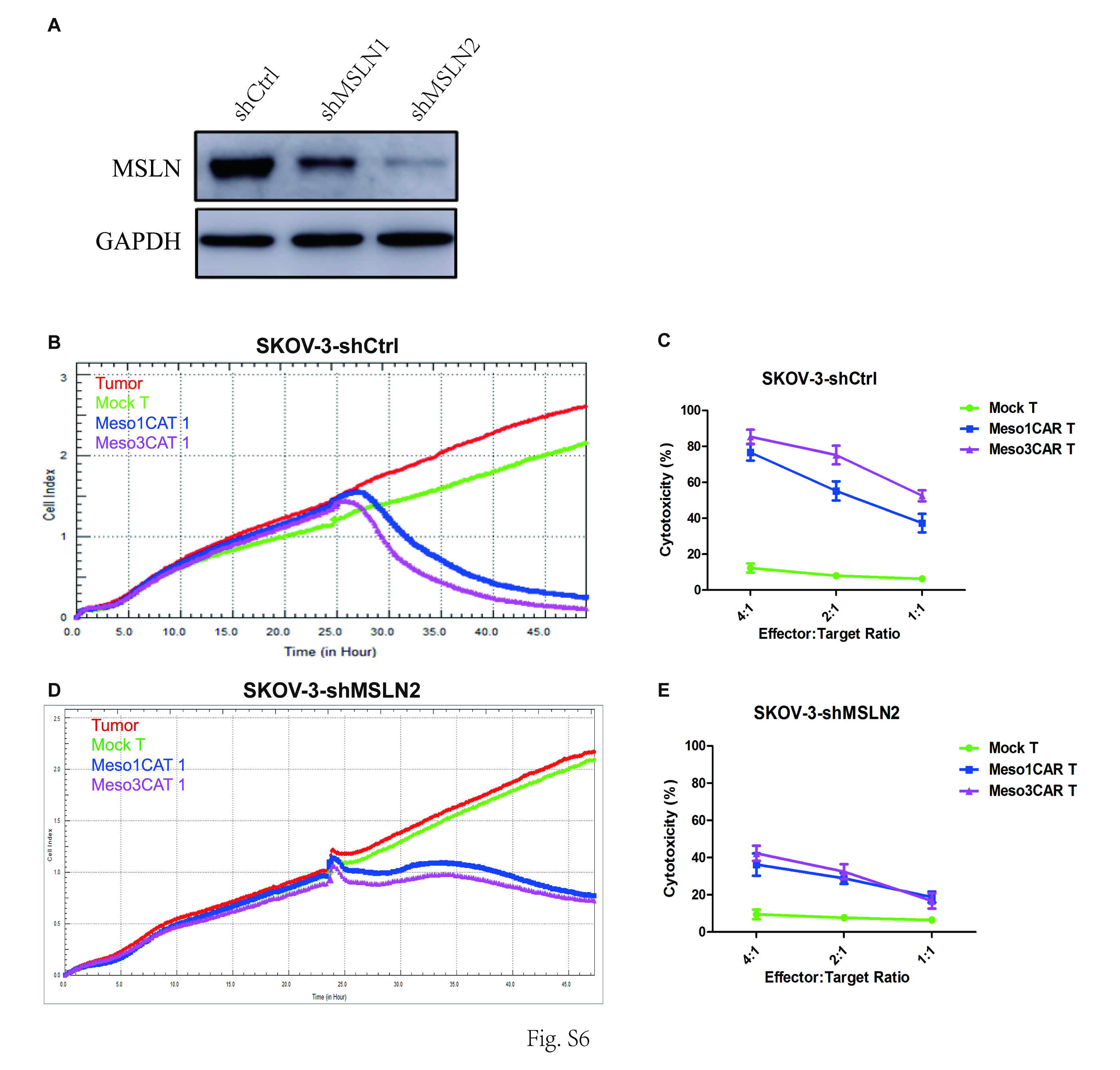

Supplement: Supplementary file 6 — Figure S6 [file 41419_2019_1711_MOESM6_ESM.tif]

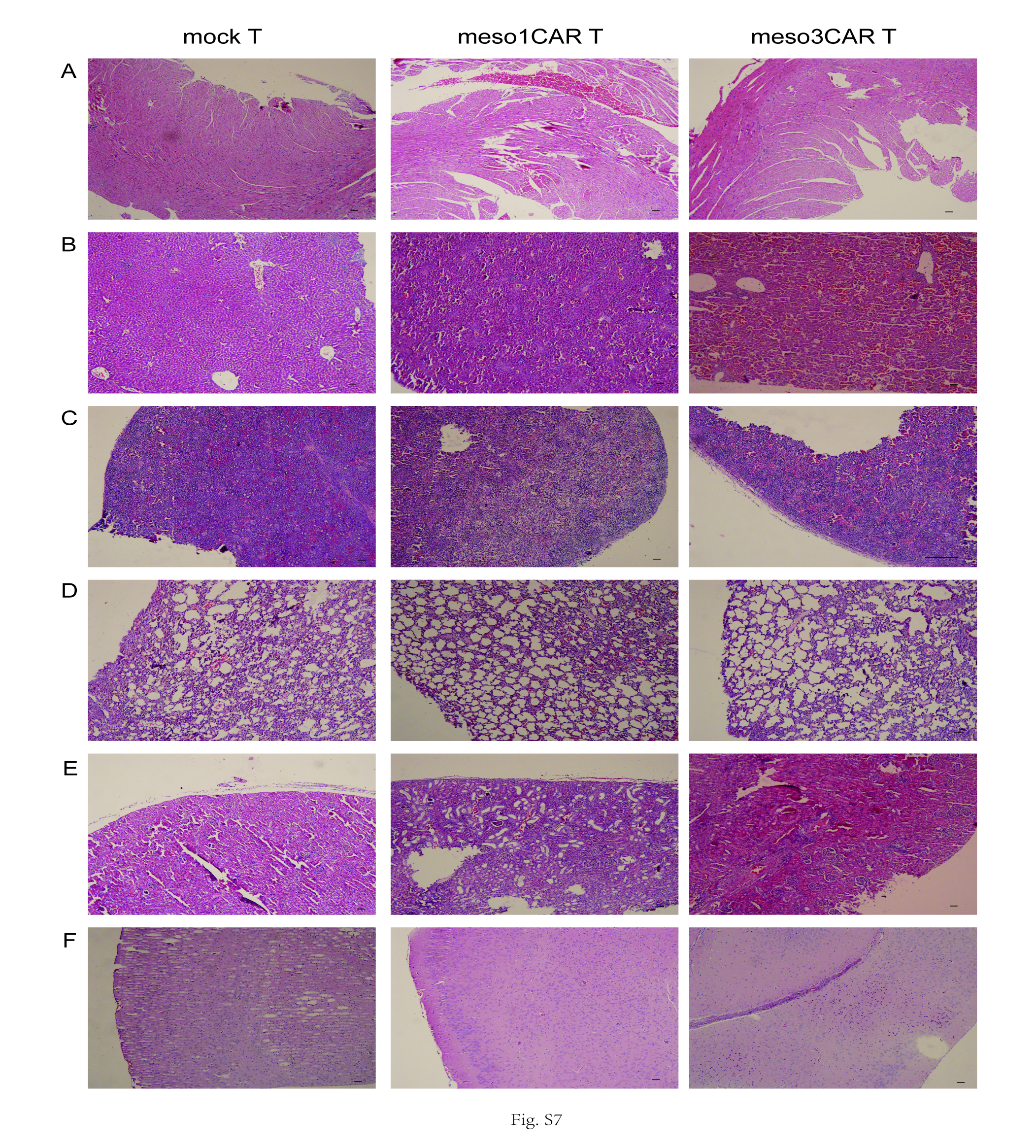

Supplement: Supplementary file 7 — Figure S7 [file 41419_2019_1711_MOESM7_ESM.tif]
