# Supplementary material for: Modified CAR T cells targeting membrane-proximal epitope of mesothelin enhances the antitumor function against large solid tumor
Source: Cell Death Dis. 2019 Jun 17;10(7):476. doi: 10.1038/s41419-019-1711-1 (PMC6572851; doi:10.1038/s41419-019-1711-1)
Supplement: Supplementary file 8 — Table S1 [file 41419_2019_1711_MOESM8_ESM.docx]

**Table S1** Expression and analysis of mesothelin in gastric cancer and ovarian cancer.

| **Characteristic** | | **No. of samples** | **No. of positive samples (%)** | ***p* value** |
| --- | --- | --- | --- | --- |
| **Gastric cancer** | |  |  |  |
|  | Meso1 antibody group | 148 | 37 (25.0) | ＜0.001 |
|  | Meso3 antibody group | 148 | 69 (46.6) |  |
| **Ovarian cancer*** | |  |  |  |
|  | Meso1 antibody group | 86 | 48 (55.8) | 0.878 |
|  | Meso3 antibody group | 86 | 47 (54.7) |  |

*Four samples were excluded because of the relatively poor stainning quality.
