# Supplementary material for: Modified CAR T cells targeting membrane-proximal epitope of mesothelin enhances the antitumor function against large solid tumor
Source: Cell Death Dis. 2019 Jun 17;10(7):476. doi: 10.1038/s41419-019-1711-1 (PMC6572851; doi:10.1038/s41419-019-1711-1)
Supplement: Supplementary file 9 — Supplementary figure legends [file 41419_2019_1711_MOESM9_ESM.docx]

**Supplementary Figure legends:**

**Fig. S1** **PD-1 expression in CAR-T cells.** (**a**) PD-1 expression in mock T cells, meso1CAR-T cells and meso3CAR-T cells were tested by flow cytometry. (**b**) Statistical analysis of the PD-1 expression in mock T cells, meso1CAR-T cells and meso3CAR-T cells. Data are expressed as the mean ± SD; n = 3; ns *p* > 0.05, *** *p <* 0.001.

**Fig. S2 T cell proliferation was analyzed by Hoechst33342/Ki-67 assay.** (**a**) Proliferation analysis in mock T cells. (**b**) Proliferation analysis in meso1CAR T cells. (**c**) Proliferation analysis in meso3CAR T cells. (**d**) Contrastive analysis of cell proliferation among mock T cells, meso1CAR T cells and meso3 CAR T cells. Ki-67^+^Hoechst^-^ represent the G1 stage and Ki-67^+^Hoechst^+^ represent the S/G2/M stage. Data are expressed as the mean ± SD; n = 3; ***p* < 0.01, *** *p* < 0.001.

**Fig. S3 The expression of MSLN was detected by IHC in the gastric cancer tissue chips.** (**a**) HE staining of gastric cancer tissue chip. (**b**) The expression of MSLN was detected using meso1 antibody in the gastric cancer tissue chip. (**c**) The expression of MSLN was detected using meso3 antibody in the gastric cancer tissue chip.

**Fig. S4 The expression of MSLN was detected by IHC in the ovarian cancer tissue chips.** (**a**) HE staining of the ovarian cancer tissue chip. (**b**) The expression of MSLN was detected using meso1 antibody in the ovarian cancer tissue chip. (**c**) The expression of MSLN was detected using meso3 antibody in the ovarian cancer tissue chip.

**Fig. S5 Representative images of MSLN expression in tissue chips of gastric cancer and ovarian cancer.** The expression of MSLN was detected in gastric cancer using IHC. (**a,b**) Representative negative staining of MSLN in gastric cancer. (**c, d**) Representative positive expression of MSLN using meso1 antibody in gastric cancer. (**e, f**) Representative positive expression of MSLN using meso3 antibody in gastric cancer. The expression of MSLN was also detected in ovarian cancer using IHC. (**g, h**) Representative negative staining of MSLN in ovarian cancer. (**i, j**) Representative positive expression of MSLN using meso1 antibody in ovarian cancer. (**k, l**) Representative positive expression of MSLN using meso3 antibody in ovarian cancer. Scale bars: 20 μm.

**Fig. S6 The cytotoxic activities of meso1CAR T cells and meso3CAR T cells in SKOV-3 cells with knockdown of MSLN.** (**a**) Mesothelin expression were tested by Western blotting in SKOV-3 cells after transfected with shMSLN or shCtrl. GAPDH was used as a loading control. (**b**) Cytotoxic activities of the meso1CAR T cells and the meso3CAR T cells against SKOV-3 cells with shCtrl were measured using RTCA system at the E:T ratio of 2:1. (**c**) Quantified data on the specific lytic levels of CAR T cells against SKOV-3 cells with shCtrl were analyzed at different E:T ratios (mean ± SD; n=3; one way ANOVA test). (**d**) Cytotoxic activities of the meso1CAR T cells and the meso3CAR T cells against SKOV-3 cells with shMSLN were measured using RTCA system at the E:T ratio of 2:1. (**e**) Quantified data on the specific lytic levels of CAR T cells against SKOV-3 cells with shMSLN were analyzed at different E:T ratios (mean ± SD; n=3; one way ANOVA test).

**Fig. S7 HE staining of normal tissue for safety analysis of meso3CAR T therapy in vivo.** Tissues from hearts (**a**), livers (**b**), spleens (**c**), lungs (**d**), kidneys (**e**), and brains (**f**) from each group were collected and paraffin-embedded. The tissue sections were stained with HE, and images were captured using a Leica DMI3000B microscope.
